# Influence of Patient-Specific Covariates on Test Validity of Two Delirium Screening Instruments in Neurocritical Care Patients (DEMON-ICU)

**DOI:** 10.1007/s12028-021-01319-9

**Published:** 2021-08-09

**Authors:** Björn Weiss, Nicolas Paul, Claudia D. Spies, Dennis Ullrich, Ingrid Ansorge, Farid Salih, Stefan Wolf, Alawi Luetz

**Affiliations:** 1 Department of Anesthesiology and Operative Intensive Care Medicine, Campus Charité Mitte and Campus Virchow-Klinikum, Charité – Universitätsmedizin Berlin, corporate member of Freie Universität Berlin and Humboldt-Universität zu Berlin, Charitéplatz 1, 10117 Berlin, Germany; 2 Department of Neurology, Charité – Universitätsmedizin Berlin, corporate member of Freie Universität Berlin and Humboldt-Universität zu Berlin, Augustenburger Platz 1, 13353 Berlin, Germany; 3 Department of Neurosurgery, Charité – Universitätsmedizin Berlin, corporate member of Freie Universität Berlin and Humboldt-Universität zu Berlin, Charitéplatz 1, 10117 Berlin, Germany; 4 Department of Healthcare Management, Technische Universität Berlin, Straße des 17. Juni, 10623 Berlin, Germany

**Keywords:** CAM-ICU, Critical care, Delirium, Hypnotics and sedatives, ICDSC, Neurocritical care, Sensitivity, Specificity

## Abstract

**Background:**

Delirium screening instruments (DSIs) should be used to detect delirium, but they only show moderate sensitivity in patients with neurocritical illness. We explored whether, for these patients, DSI validity is impacted by patient-specific covariates.

**Methods:**

Data were prospectively collected in a single-center quality improvement project. Patients were screened for delirium once daily using the Intensive Care Delirium Screening Checklist (ICDSC) and the Confusion Assessment Method for the Intensive Care Unit (CAM-ICU). Reference was the daily assessment using criteria from the Diagnostic and Statistical Manual, 4th Edition, Text Revision (DSM-IV-TR). In a two-step receiver operating characteristics regression analysis adjusting for repeated measurements, the impact of acute diagnosis of stroke or transient ischemic attack (TIA), neurosurgical intervention, Richmond Agitation Sedation Scale, and ventilation status on test validity was determined.

**Results:**

Of 181 patients screened, 101 went into final analysis. Delirium incidence according to DSM-IV-TR was 29.7%. For the first complete assessment series (CAM-ICU, ICDSC, and DSM-IV-TR), sensitivity for the CAM-ICU and the ICDSC was 73.3% and 66.7%, and specificity was 91.8% and 94.1%, respectively. Consideration of daily repeated measurements increased sensitivity for the CAM-ICU and ICDSC to 75.7% and 73.4%, and specificity to 97.3% and 98.9%, respectively. Receiver operating characteristics regression revealed that lower Richmond Agitation Sedation Scale levels significantly impaired validity of the ICDSC (*p* = 0.029) and the CAM-ICU in its severity scale version (*p* = 0.004). Neither acute diagnosis of stroke or TIA nor neurosurgical intervention or mechanical ventilation significantly influenced DSI validity.

**Conclusions:**

The CAM-ICU and ICDSC perform well in patients requiring neurocritical care, regardless of the presence of acute stroke, TIA, or neurosurgical interventions. Yet, even very light or moderate sedation can significantly impair DSI performance.

**Supplementary Information:**

The online version contains supplementary material available at 10.1007/s12028-021-01319-9.

## Introduction

Delirium is the most typical manifestation of a *per-definition* secondary encephalopathy in the critical care context [1]. Studies revealed that up to 82% of mechanically ventilated patients suffer from delirium [1]. Because delirium impairs outcomes [1–3], regular screening with a validated delirium screening instrument (DSI) is recommended in national and international guidelines, with a high level of evidence [4, 5]. DSIs such as the Confusion Assessment Method for the Intensive Care Unit (CAM-ICU) [6] and the Intensive Care Delirium Screening Checklist (ICDSC) [7] have shown a sufficient diagnostic validity and reliability in medical and surgical intensive care and are considered to be the standard for the diagnosis of delirium [8].

There are only a few studies pertaining to delirium in patients with neurocritical illness. These patients show a pooled delirium prevalence rate of 11.8% to 45.9% in prospective cohort studies [9]. Just like in other patient populations, delirium in this cohort is associated with increased intensive care unit (ICU) and hospital length of stay, cost of care, and occurrence of postintensive care syndrome, particularly long-term cognitive impairments [9].

Neurological and neurosurgical patients typically show symptoms that, by their nature, are related to the patient’s primary pathology (e.g., edema, seizures, or ischemia). These symptoms could potentially alter and overlap with symptoms that are assessed during a delirium screening and therefore lead to a false positive DSI result and delayed diagnosis of the underlying problem. At worst, exacerbation of the underlying disease could be misinterpreted as delirium. In a meta-analysis, Patel et al. [9] rightfully caution their readership that information from a delirium screening should only be considered complementary to the neurological examination, which is paramount to diagnose complex complications. Therefore, validation of the DSIs in the context of neurocritical care is of utmost importance. Previously, the diagnostic validity of the CAM-ICU and the ICDSC in patients with neurological and neurosurgical critical illness was assessed in five studies that revealed a moderate sensitivity and a good to excellent specificity [10–14]. Yet, no study to date has investigated whether the Richmond Agitation Sedation Scale (RASS), mechanical ventilation, presence of an acute stroke or a transient ischemic attack (TIA), and neurosurgical interventions directly impact the diagnostic validity of the CAM-ICU and the ICDSC in patients with neurocritical illness.

## Methods

### Project Setting and Design

We report on a quality improvement (QI) project conducted at Charité – Universitätsmedizin Berlin. Data were prospectively collected as part of internal QI procedures, as part of delirium assessment according to the hospital’s standard operating procedure (SOP), and according to a national guideline. Approval of data protection was obtained before data collection. Scientific publication of results was approved by the Ethics Committee at Charité – Universitätsmedizin Berlin (EA1/228/15) post-hoc and written informed consent was waived. The research has been carried out in accordance with the Declaration of Helsinki from 1964 and its amendments.

### Patients

We consecutively enrolled patients with critical illness admitted to one neurological/neurosurgical ICU at Charité – Universitätsmedizin Berlin between 15 September 2011 and 14 October 2011 (first assessment period) and between 1 February and 14 April 2012 (second assessment period). Exclusion criteria of the QI project were as follows: Age below 18 years, lack of German proficiency, clinically manifest dementia or other psychiatric disorder, lack of willingness to participate in assessments, and patient likely to pass away in the subsequent 24 h.

### Delirium Assessments

**Fig. 1:**
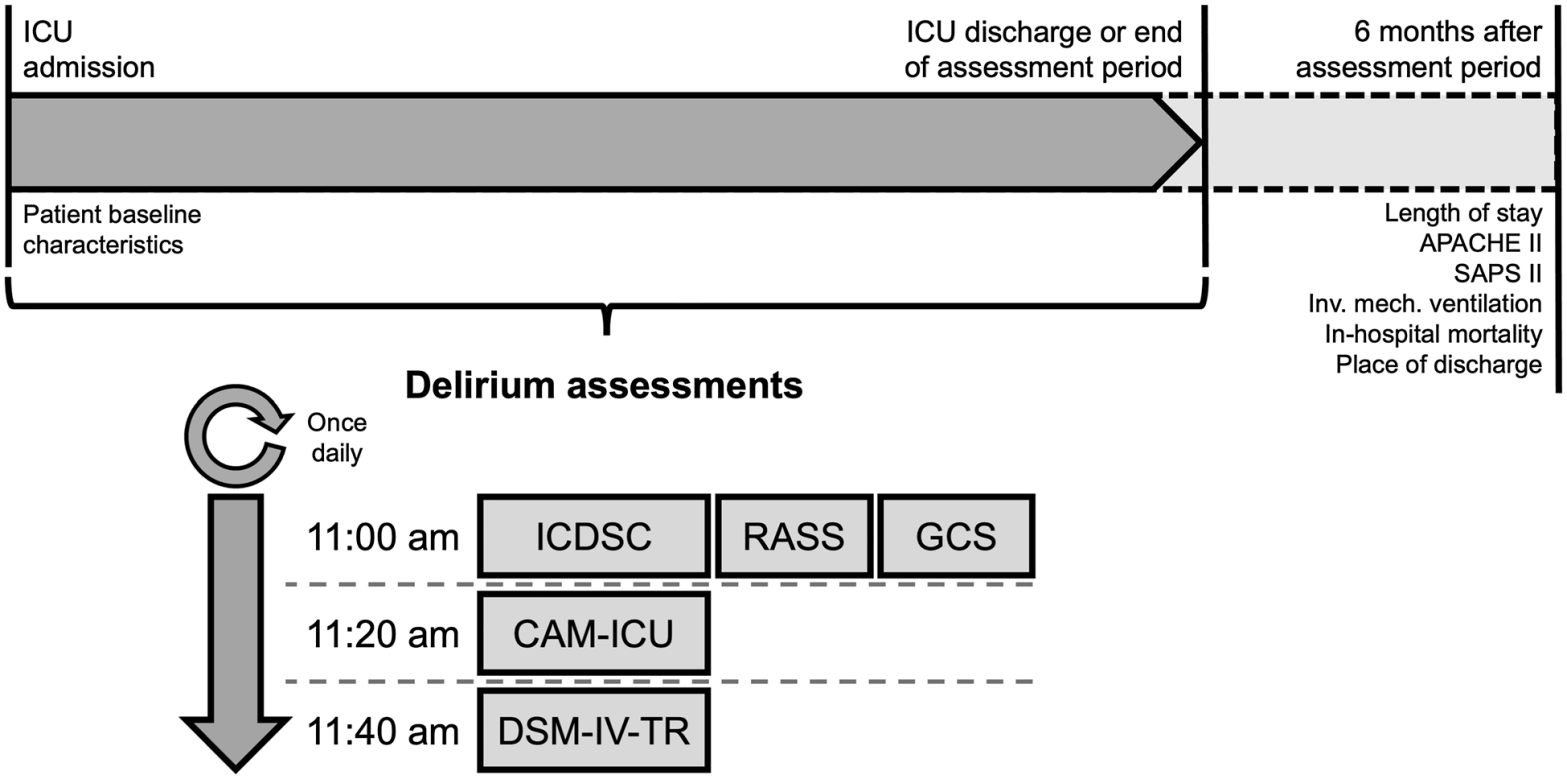
Delirium assessment and data collection schedule. *APACHE II* Acute Physiology and Chronic Health Disease Classification System II, *CAM-ICU* Confusion Assessment Method for the Intensive Care Unit, *DSM-IV-TR* Diagnostic and Statistical Manual, 4th Edition, Text Revision, *GCS* Glasgow Coma Scale, *ICDSC* Intensive Care Delirium Screening Checklist, *ICU*, intensive care unit, *Inv. mech. ventilation* Invasive mechanical ventilation, *RASS* Richmond Agitation Sedation Scale, *SAPS II*, Simplified Acute Physiology Score II

Patients were assessed for delirium once daily using the ICDSC and the CAM-ICU from the day of ICU admission until discharge or end of assessment period (Fig. 1). As a reference standard, Diagnostic and Statistical Manual, 4th Edition, Text Revision (DSM-IV-TR) assessment was conducted once daily. Independent from routine care, ICDSC and CAM-ICU were assessed by specially trained QI project staff (DU, among others) under the supervision of experienced intensivists (AL, among others). DSM-IV-TR was assessed by specially trained QI project staff under supervision of an intensivist who was backed up by a board-certified psychiatrist. Assessors received training as previously described [15]. Briefly, training comprised oral presentations, printed information material, hands-on application, and reference testing of five consecutive patients who had previously been tested by two experienced intensivists. Differences between assessors and experienced intensivists were discussed to reach consensus [15]. DSIs and DSM-IV-TR were always applied at the same time of the day and in the same order (Fig. 1). To avoid bias due to between-observer variation, each assessment tool was consistently applied by one assessor. To ensure that each assessor was blinded to the results of the other assessors, there were gaps of 20 min between delirium assessments. All assessments were conducted within 90 min to avoid fluctuations of symptoms. Daily and in synchrony with delirium assessments, vigilance [Glasgow Coma Scale (GCS)] and level of sedation (RASS) were documented. The RASS was used for intubated and nonintubated patients. If a patient was comatose or deeply sedated (RASS <  − 3 or GCS < 9), no delirium assessment was conducted on that particular occasion. Immediately after assessments, results were electronically documented with a patient-specific pseudonym using LimeSurvey (LimeSurvey GmbH, Hamburg, Germany). Patients were excluded from analysis if, due to deep sedation or coma, no complete assessment series (ICDSC, CAM-ICU, and DSM-IV-TR) could be conducted until discharge or end of assessment period. Results of the CAM-ICU were transformed from a binary to an ordinal scale as previously described for the transformation of the pediatric CAM-ICU [16]. Accordingly, the ordinal CAM-ICU was named severity scale for the CAM-ICU (ssCAM-ICU).

### Assessment of Patient Characteristics

On project enrollment, patients’ age, sex, weight, reason of admission (including neurosurgical intervention), main International Classification of Diseases, 10th revision (ICD-10), diagnosis (including acute diagnosis of stroke/TIA), history of clinically manifest dementia, diagnosis of alcohol use disorder, and history of stroke or TIA were documented. Six months after the assessment period, the following data were extracted from the patients’ electronic medical record: ICU length of stay, presence and duration of invasive mechanical ventilation, Acute Physiology and Chronic Health Disease Classification System II (APACHE II) and Simplified Acute Physiology Score II (SAPS II) scores upon admission, in-hospital mortality, and place of discharge.

### Statistical Analysis

Descriptive analysis of the QI project population with corresponding distributions is presented as either median with limits of the interquartile range (25th to 75th percentile) or as absolute (*n*) or relative (%) frequencies. Differences in characteristics between the group of patients who were delirium-positive according to DSM-IV-TR in at least one assessment and the group of patients who were delirium-negative were compared using the Mann–Whitney *U*-test for continuous variables, Fisher’s exact test for frequencies with two categories, and *χ*^2^ test for frequencies with three or more categories. Using each patient’s first complete assessment series, sensitivity, specificity, positive predictive value (PPV), and negative predictive value (NPV) were calculated and compared using McNemar test. The first complete assessment series was defined as the first time all assessment tools (ICDSC, CAM-ICU, and DSM-IV-TR) were applied. Empirical receiver operating characteristics (ROC) curves of the ICDSC and ssCAM-ICU were plotted for the first complete assessment series using the DSM-IV-TR as the binary classifier for delirium, area under the curve (AUC) determined and compared according to DeLong et al. [17]. Including all assessments of the ICDSC and the CAM-ICU, we fitted a logistic regression model with random effects to determine sensitivity, specificity, PPV and NPV, adjusting for repeated measurements per patient (binary clustered data) [18–20]. All assessments were defined as any assessment pair of DSM-IV-TR and ICDSC or CAM-ICU, respectively. Empirical ROC curves were plotted for all assessments of the ICDSC and ssCAM-ICU using the DSM-IV-TR as the binary classifier for delirium, AUCs determined and compared using Somer’s D and Harrell’s c, adjusting for repeated assessments [21, 22]. Pietra indexes were calculated for the ROC curves. The Pietra index describes a test’s ability to distinguish between patients who were delirium-positive and delirium-negative and has a range of 0 (no distinction between patients who were delirium-positive and delirium-negative) and 1 (perfect distinction between patients who were delirium-positive and delirium-negative) [23]. We assessed the influence of the following covariates on DSI validity: RASS score, mechanical ventilation (yes/no), sex (male/female), neurosurgical intervention (yes/no), and acute diagnosis of stroke/TIA (yes/no). Acute diagnosis of stroke/TIA and neurosurgical intervention (which included surgical procedures only) were selected because they resemble common and distinct patient cohorts in neurocritical care. Patients who underwent neurosurgical interventions are commonly treated in non-neurological ICUs, whereas patients with acute stroke/TIA are commonly treated in specialized stroke units or neurological ICUs. To assess the effect of the covariates on DSI validity, we performed a two-step ROC regression analysis [24], defining covariates for the control population (DSM-IV-TR negative) and case population (DSM-IV-TR positive), including all assessments and adjusting for repeated assessments. To confirm ROC regression results, empirical, covariate-adjusted ROC curves were plotted and AUCs determined. Analysis was performed with STATA 13.1 (StataCorp LP, College Station, TX).

## Results

### Characteristics of the Project Cohort

**Fig. 2:**
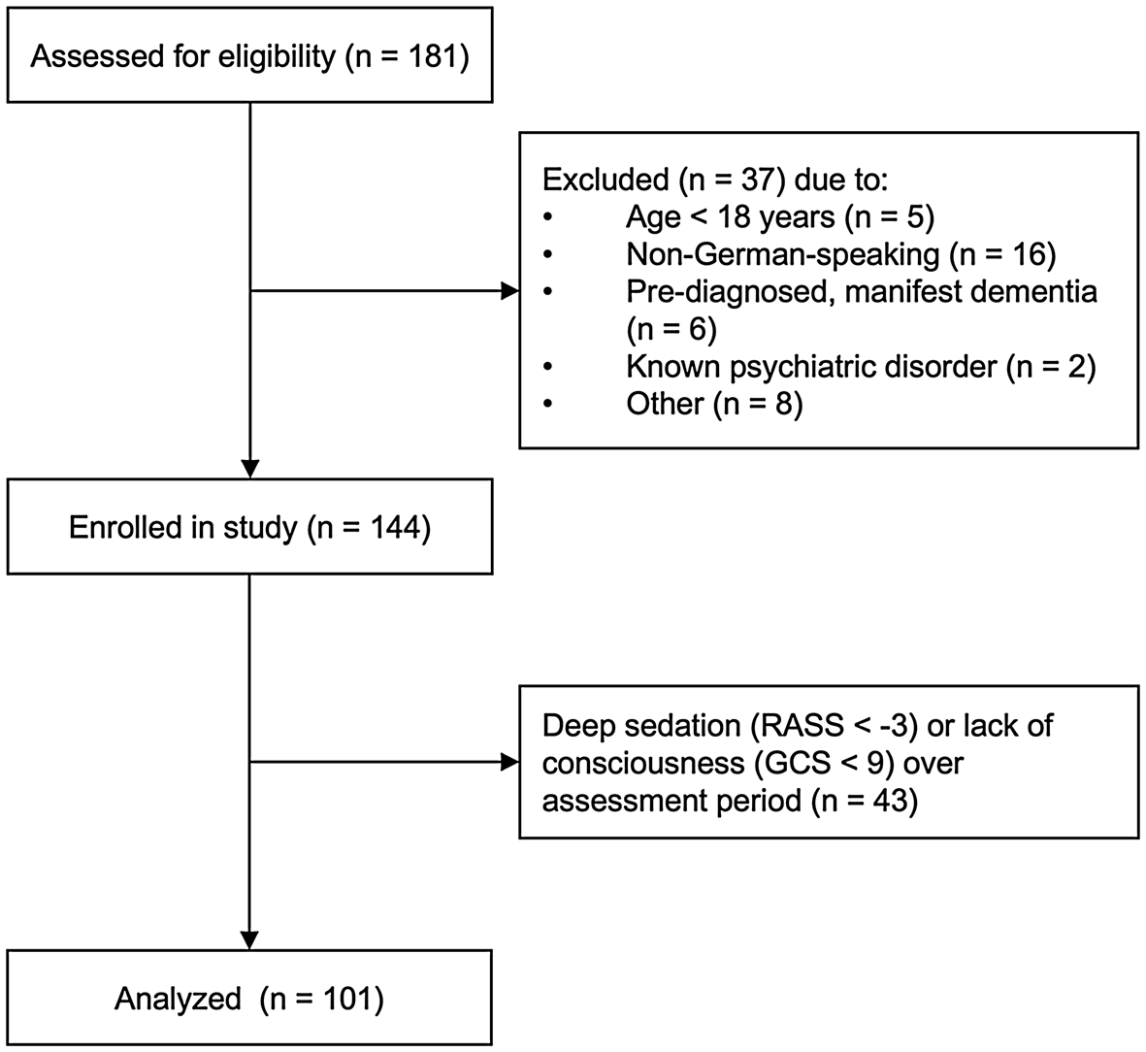
Consort diagram. Other includes: patient refused to participate in assessments, patient likely to pass away in the following 24 h, and patient discharged from ICU before assessment of all scores. *GCS* Glasgow Coma Scale, *ICU* Intensive care unit, *RASS* Richmond Agitation Sedation Scale

Out of *n* = 181 patients screened to participate in the QI project, *n* = 80 (44.2%) were excluded, leaving *n* = 101 (55.8%) patients for analysis (Fig. 2). Baseline characteristics are described in Table 1. Thirty (29.7%) patients were tested positive for delirium according to DSM-IV-TR on at least one occasion. Patients in the delirium group showed a higher severity of illness [APACHE II on admission 20 (16–26) vs. 13 (7–19), *p* < 0.001; SAPS II on admission 39.5 (31–49) vs. 25.5 (15–38), *p* < 0.001]. Significantly more patients in the delirium group were mechanically ventilated [*n* = 22 (73.3%) vs. *n* = 22 (31.0%), *p* < 0.001] with longer duration [105.5 (0–185) vs. 0 (0–11) hours, *p* < 0.001]. Patients in the delirium group had a longer ICU length of stay [15.5 (9–22) vs. 3 (1–6) days, *p* < 0.001], higher in-hospital mortality [*n* = 5 (16.7%) vs. *n* = 0 (0%), *p* = 0.002] and spent more days with sedation [1.5 (0–5) vs. 0 (0–0), *p* < 0.001]. Sedation was defined as at least light sedation (RASS − 2 or lower) during the assessment.

**Table 1:** Descriptive statistics of the study population

Variable	Delirium^a^ (*n* = 30)	No delirium^a^ (*n* = 71)	*p*
Age^b^ (yr)	64.5 (58–75)	60 (50–76)	0.506^i^
Male sex, *n*	20	34	0.126^j^
Weight^b^ (kg)	78 (70–85)	71 (65–81)	0.160^i^
APACHE II on admission^b^	20 (16–26)	13 (7–19) (*n* = 67)	< 0.001^i^
SAPS II on admission^b^	39.5 (31–49)	25.5 (15–38) (*n* = 70)	< 0.001^i^
Reason of admission, *n*			
Neurosurgical intervention	17	42	0.530^k^
Hemodynamics/cardiology^c^	2	1	
Neurology	10	24	
Surgery (other than neurosurgery)^d^	1	4	
Patients underwent CPR, *n*	1	1	0.508^j^
Patients with known stroke or TIA, *n*	8	30	0. 179^j^
Patients with a diagnosis of alcohol use disorder, *n*	4	5	0.311^j^
Main ICD-10 diagnosis, *n*			
Injuries of the head	5	9	0.514^k^
Neoplasms of unknown behavior	0	6	
Cerebrovascular disease (stroke/TIA)	14	33	
Malignant neoplasms	5	8	
Others^e^	6	15	
In-hospital mortality, *n*	5	0	0.002^j^
Discharged to other ICU, *n*	13	13	0.013^j^
Patients receiving invasive mechanical ventilation^f^, *n*	22	22	< 0.001^j^
Invasive mechanical ventilation^b^ (h)	105.5 (0–185)	0 (0–11)	< 0.001^i^
ICU length of stay^b^ (d)	15.5 (9–22)	3 (1–6)	< 0.001^i^
Sedation^b,g^ (d)	1.5 (0–5)	0 (0–0)	< 0.001^i^
Time in delirium^b,h^ (d)	1.5 (1–2)	0 (0–0)	< 0.001^i^

*APACHE II* Acute Physiology and Chronic Health Disease Classification System II, *CPR* Cardiopulmonary resuscitation, *DSM-IV-TR* Diagnostic and Statistical Manual, 4th Edition, Text Revision, *ICD-10* International Classification of Diseases 10th revision, *ICU* Intensive care unit, *SAPS II* Simplified Acute Physiology Score II, *TIA* Transient ischemic attack

^a^Delirium on at least one occasion during assessment period according to DSM-IV-TR

^b^Data presented as median (limits of the interquartile range)

^c^Out of the three patients admitted to the ICU because of hemodynamic instability, one patient was treated in the hospital for stroke, one patient for meningitis, and one patient for intracerebral hemorrhage

^d^Out of the five patients admitted to the ICU because of surgery (other than neurosurgery), four patients had polytrauma, including traumatic brain injury, and one patient had a stroke during ICU treatment after orthopedic surgery

^e^Supplement 1 lists diagnoses of patients grouped as others

^f^Patients receiving mechanical ventilation on at least one occasion during the study period

^g^Days spent with RASS <  − 1

^h^Number of days with delirium according to DSM-IV-TR

^i^Mann–Whitney *U*-test

^j^Fisher’s exact test

^k^*χ*^2^ test

### Validity of the ICDSC, CAM-ICU, and ssCAM-ICU for the First Complete Assessment Series

**Fig. 3:**
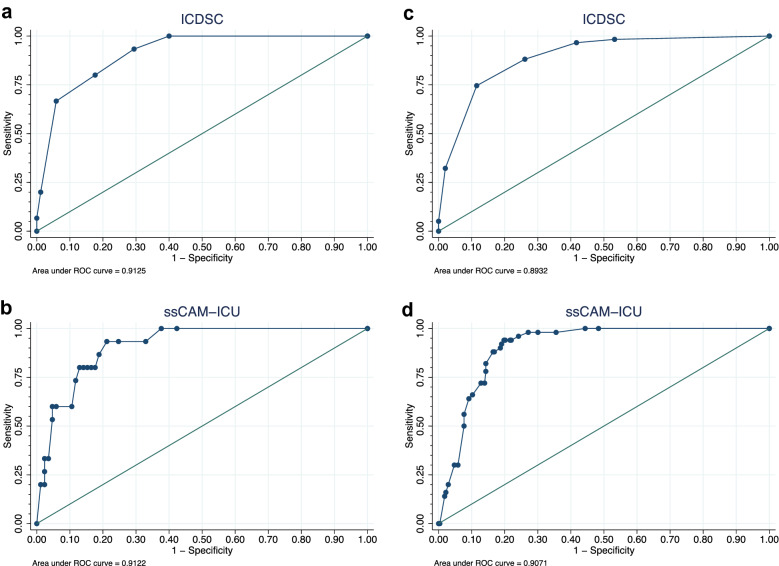
ROC curves of the ICDSC and ssCAM-ICU for the first complete assessment series (**a**, **b**) and for all assessments (**c**, **d**). The first complete assessment series was defined as first time all assessment tools (ICDSC, CAM-ICU, and DSM-IV-TR) were applied. All assessments were defined as any assessment pair of DSM-IV-TR and ICDSC or CAM-ICU, respectively. The DSM-IV-TR was used as reference for delirium assessments. *CAM-ICU* Confusion Assessment Method for the Intensive Care Unit, *DSM-IV-TR* Diagnostic and Statistical Manual, 4th Edition, Text Revision, *ICDSC* Intensive Care Delirium Screening Checklist, *ROC* Receiver operating characteristic, *ssCAM-ICU* Severity scale Confusion Assessment Method for the Intensive Care Unit

In the first complete assessment series, *n* = 15 (15%) patients were diagnosed positive for delirium according to DSM-IV-TR. The CAM-ICU showed a sensitivity of 73.3%, a specificity of 91.8%, a PPV of 61.1%, and a NPV of 95.1%. The ICDSC had a sensitivity of 66.7%, a specificity of 94.1%, a PPV of 66.7%, and a NPV of 94.1% (Table 2). Test validities of the CAM-ICU and the ICDSC were not significantly different (*p* = 0.317). As shown in Fig. 3a, b, ROC analysis revealed an AUC of 0.913 for the ICDSC and 0.912 for the ssCAM-ICU, with no significant difference (*p* = 0.993). The Pietra index was 0.50 for the ICDSC and 0.39 for the ssCAM-ICU. The difference in Pietra indexes indicates that the ICDSC has a better ability to distinguish between patients who were delirium-positive and delirium-negative.

**Table 2:** Test validity of the CAM-ICU and ICDSC for the first complete assessment series^a^ per patient

Delirium screening instrument	Sensitivity, % (95% CI)	Specificity, % (95% CI)	Positive predictive value, % (95% CI)	Negative predictive value, % (95% CI)
CAM-ICU (*n* = 100)^b^	73.3 (44.9–92.2)	91.8 (83.8–96.6)	61.1 (35.7–82.7)^c^	95.1 (88.0–98.7)^c^
ICDSC (*n* = 100)^b^	66.7 (38.4–88.2)	94.1 (86.8–98.1)	66.7 (38.4–88.2)^c^	94.1 (86.8–98.1)^c^

*CAM-ICU* Confusion Assessment Method for the Intensive Care Unit, *CI* Confidence interval, *DSM-IV-TR* Diagnostic and Statistical Manual, 4th Edition, Text Revision, *ICDSC* Intensive Care Delirium Screening Checklist, ICU Intensive care unit

^a^First complete assessment series defined as first time all assessment tools (ICDSC, CAM-ICU, and DSM-IV-TR) were applied

^b^Comparison of test validities of CAM-ICU and ICDSC using McNemar test: *p* = 0.317. One patient was uncooperative during CAM-ICU assessment. Thus, there was no first complete assessment series for this patient

^c^Delirium prevalence for the first assessment series was *n* = 15 (15.0%)

### Validity of the ICDSC, CAM-ICU, and ssCAM-ICU for all Assessments

For the CAM-ICU, *n* = 324 corresponding DSM-IV-TR and CAM-ICU assessments were conducted, and for the ICDSC, *n* = 346 corresponding DSM-IV-TR and ICDSC assessments were conducted. For the CAM-ICU, the logistic regression model with random effects revealed a sensitivity of 75.7%, a specificity of 97.3%, a PPV of 52.8%, and a NPV of 98.9% (Table 3). For the ICDSC, the model revealed a sensitivity of 73.4%, a specificity of 98.9%, a PPV of 61.2%, and a NPV of 94.6%. Comparison of test validities showed no significant differences. ROC analysis yielded an AUC of 0.893 for the ICDSC and 0.907 for the ssCAM-ICU (Fig. 3c, d) with no significant differences (*p* = 0.465). The ability of the DSIs to distinguish between patients who were delirium-positive and delirium-negative as measured with the Pietra index was 0.62 for the ICDSC and 0.46 for the ssCAM-ICU. Just like for the first complete assessment series, the difference in Pietra indexes indicates that the ICDSC has a better ability to distinguish between patients who were delirium-positive and delirium-negative.

**Table 3:** Test validity of the CAM-ICU and ICDSC for all assessments^a^ employing a logistic regression model with random effects

Delirium screening instrument	Sensitivity, % (95% CI)	Specificity, % (95% CI)	Positive predictive value, % (95% CI)	Negative predictive value, % (95% CI)
CAM-ICU (*n* = 324)	75.7 (55.9–88.5)^b^	97.3 (89.9–99.3)^c^	52.8 (36.7–68.3)^d^	98.9 (89.9–99.9)^e^
ICDSC (*n* = 346)	73.4 (59.1–84.1)^b^	98.9 (93.4–99.8)^c^	61.2 (44.2–75.9)^d^	94.6 (85.4–98.1)^e^

^a^All assessments defined as any assessment pair of DSM-IV-TR and ICDSC or CAM-ICU, respectively

*CAM-ICU* Confusion Assessment Method for the Intensive Care Unit, *CI* Confidence interval, *DSM-IV-TR* Diagnostic and Statistical Manual, 4th Edition, Text Revision, *ICDSC* Intensive Care Delirium Screening Checklist

^b^Significance level of impact of test type on sensitivity: *p* = 0.999

^c^Significance level of impact of test type on specificity: *p* = 0.057

^d^Significance level of impact of test type on positive predictive value: *p* = 0.329

^e^Significance level of impact of test type on negative predictive value: *p* = 0.746

### Impact of Patient-Specific Covariates on the Validity of ICDSC and ssCAM-ICU

**Fig. 4:**
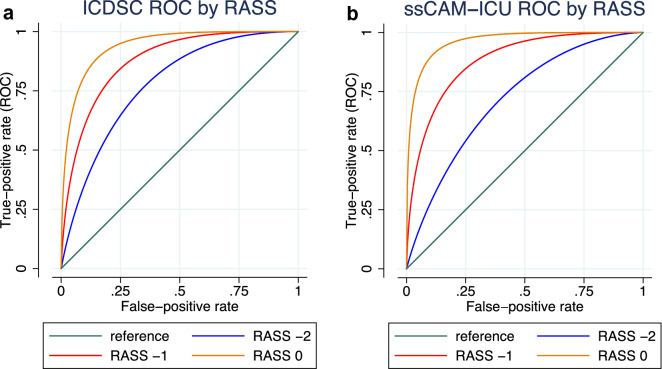
ROC regression analysis for the ICDSC (**a**) and ssCAM-ICU (**b**) for different RASS scores, including all assessments. All assessments were defined as any assessment pair of DSM-IV-TR and ICDSC or CAM-ICU, respectively. *DSM-IV-TR* Diagnostic and Statistical Manual, 4th Edition, Text Revision, *ICDSC* Intensive Care Delirium Screening Checklist, *RASS* Richmond Agitation Sedation Scale, *ROC* Receiver operating characteristic, *ssCAM-ICU* Severity scale Confusion Assessment Method for the Intensive Care Unit

As shown in Tables 4 and 5, the parametric ROC regression model revealed that only the RASS score had a significantly positive effect on the ROC curve of the ICDSC (coefficient 0.634, *p* = 0.029), and the ssCAM-ICU (coefficient 0.920, *p* = 0.004). Thus, higher RASS scores were associated with better test validities of the ICDSC and the ssCAM-ICU. Figure 4 illustrates ROC curves for different RASS scores and shows that for both DSIs, AUCs of ROC curves for sedated patients (RASS − 2) are smaller than for nonsedated patients (RASS − 1 or 0). Neither a neurosurgical intervention nor an acute diagnosis of stroke/TIA had a significant impact on the ROC curves of the ssCAM-ICU or the ICDSC. Results were confirmed by plotting empirical ROC curves for covariates, as depicted in Supplement 2. AUCs of the empirical ROC curves for patients with a RASS score of 0 or − 1 were greater than AUCs of the empirical ROC curves for patients with a RASS score outside this range, whereas AUCs of empirical ROC curves of other covariates, including neurosurgical intervention and acute diagnosis of stroke/TIA, did not show great differences.

**Table 4:** ROC regression analysis for the ICDSC under the influence of different covariates

Covariate	ROC coefficient (95% CI)	*p*
RASS score	0.634 (0.064–1.204)	0.029
Sex (male/female)	0.023 (− 0.632–0.677)	0.945
Mechanical ventilation (yes/no)	− 0.235 (− 0.669–0.199)	0.289
Neurosurgical intervention (yes/no)	0.473 (− 0.304–1.250)	0.232
Acute diagnosis of stroke/TIA (yes/no)	− 0.060 (− 0.593–0.472)	0.825

The nonparametric covariate control adjustment model showed that RASS score (*p* = 0.002) and neurosurgical intervention (*p* = 0.029) had a significant effect on the ICDSC under the control population. Hence, the ROC regression model was fit by employing RASS score and neurosurgical intervention as covariates for the control population and RASS score, sex, mechanical ventilation, neurosurgical intervention, and acute diagnosis of stroke/TIA as covariates for the case population. RASS score had a significant positive effect on the ROC curve

*CI* Confidence interval, *ICDSC* Intensive Care Delirium Screening Checklist, *RASS* Richmond Agitation Sedation Scale, *ROC* Receiver operating characteristic, *TIA* Transient ischemic attack

**Table 5:** ROC regression analysis for the ssCAM-ICU under the influence of different covariates

Covariate	ROC coefficient (95% CI)	*p*
RASS score	0.920 (0.297–1.543)	0.004
Sex (male/female)	0.347 (− 0.308–1.001)	0.299
Mechanical ventilation (yes/no)	0.589 (− 0.102–1.280)	0.095
Neurosurgical intervention (yes/no)	0.166 (− 0.587–0.919)	0.665
Acute diagnosis of stroke/TIA (yes/no)	− 0.027 (− 0.710–0.655)	0.938

The nonparametric covariate control adjustment model showed that RASS score had a significant effect on the ssCAM-ICU under the control population (*p* < 0.001). Hence, the ROC regression model was fit by employing RASS score as a covariate for the control population and RASS score, sex, mechanical ventilation, neurosurgical intervention, and acute diagnosis of stroke/TIA as covariates for the case population. RASS score had a significant positive effect on the ROC curve

*CI* Confidence interval, *RASS* Richmond Agitation Sedation Scale, *ROC* Receiver operating characteristic, *ssCAM-ICU* Severity Scale Confusion Assessment Method for the Intensive Care Unit, *TIA* Transient ischemic attack

## Discussion

In this single-center QI project, we prospectively assessed the diagnostic validity of the most common DSIs CAM-ICU and ICDCS in a neurocritical care population. Taking into account single and multiple testing on one patient, we revealed a moderate to good sensitivity and an excellent specificity in this patient cohort. In a second step, we examined the influence of neurocritical care-specific and general covariates on the diagnostic validities of the CAM-ICU and ICDSC, which revealed a significant influence of the RASS score on the diagnostic performance of the DSIs. Neither acute diagnosis of stroke/TIA or neurosurgical intervention, nor the general covariates sex, age, or mechanical ventilation had a significant impact on DSI validities.

The delirium rate of 29.7% found in this QI project lies within the expected range in patients with neurocritical illness. Previous trials reported a delirium prevalence ranging from 11.8 to 45.9% [10–12, 25–27]. Variations are most likely because of different compositions of the study populations. For example, Patel et al. [9] found in a systematic review that the incidence of mechanical ventilation varied from less than 10% to more than 60% between studies, and the relationship between ventilator status, severity of illness and prevalence of delirium has previously been described [1].

Regarding the diagnostic validity of the DSIs, our findings are in line with a recently published observational study of 123 neurocritical care patients [14], which revealed a sensitivity and specificity of 66.9% and 93.3% for the CAM-ICU, as well as 69.9% and 93.9% for the ICDCS. In that study, the ICD-10 was used as a reference standard, which is known to be less sensitive in diagnosing delirium than the DSM-IV-TR that was used in our QI project [28]. In addition, the authors report a significant association between a positive DSI and the presence of neurological symptoms, which implies that a positive delirium screening should lead to further neurological diagnostics to delineate if the patient has delirium or another neurological condition. Contrary to their results, our data suggest that the presence of acute stroke or TIA does not diminish diagnostic validity of the DSIs. In fact, the specificity of both DSIs remains excellent.

Patel et al. [9] identified four additional studies that assessed the validity of different DSIs versus a reference standard in patients with neurocritical illness [10–13]. The CAM-ICU and the ICDSC were most commonly used as DSIs. Two studies using the CAM-ICU against the reference standard DSM-IV and DSM-IV-TR, respectively, revealed a sensitivity of 76.0% and 62%, and a specificity of 98.1% and 74%, respectively [10, 12]. One study that used the ICDSC against the DSM-IV-TR as a reference standard revealed a sensitivity of 64% and a specificity of 79% [10]. Compared with these studies, our data suggest a comparable or slightly better diagnostic validity. The most recently published data in a medical/surgical critical care population exhibit comparable diagnostic validities for the ICDSC and the CAM-ICU [29]. Our observation that diagnostic validity slightly increases if one accounts for multiple testing is also a consistent finding among studies with medical/surgical patients with critical illness [15, 29].

This work adds to current literature that DSI validity is influenced by the same covariates in neurocritical care patients as in medical/surgical critical care patients [15, 29], namely the RASS score. We do not see an effect of neurocritical care-specific covariates, which allows for the conclusion that scores remain valid despite neurological diagnoses or neurosurgical procedures, which contrasts previous findings [14]. However, the number of potential differential diagnoses for a positive DSI in neurocritical care patients exceeds the number of differential diagnoses in medical/surgical ICU patients. This means that diagnoses such as nonconvulsive seizures and intracerebral pathologies are generally observed more frequently compared with the medical/surgical ICU context. This underscores that clinicians should refrain from using DSIs without a full neurological examination to determine whether a patient suffers from delirium or a primary encephalopathy, leading to a false positive DSI result.

This QI project has several limitations. Our patient cohort consisted of a heterogeneous group of patients with neurocritical illness. On the one hand, this can be considered a strength as it does not limit the results to one specific neurocritical care patient subgroup, on the other hand, our study might underestimate the impact of covariates in individual subgroups. Because the project results were in line with previous works, this limitation might be mitigated. In addition, a larger sample size would have increased the precision of our estimates and the statistical power to detect a significant influence of covariates on DSI validity. However, our sample size was comparable to other DSI validation studies [14–16, 29]. Furthermore, no detailed neurological examination was performed, which could have identified neurological symptoms or syndromes that show a similar appearance to delirium and therefore cause false positive DSI results. Future studies should therefore take a standardized neurological status to delineate delirium and primary encephalopathies. Ultimately, this is a single-center QI project, which limits its generalizability because center-related effects, such as context-specific SOPs, might influence our results.

## Conclusions

In summary, delirium screening in patients with neurocritical illness performs with an adequate diagnostic validity, irrespective of the presence of acute stroke, TIA, or neurosurgical interventions. However, even a very light sedation level significantly impairs DSI performance. Notably, a false positive DSI in neurocritical care patients might be due to different differential diagnoses than that in the medical or surgical critical care population.

## Supplementary Information

Below is the link to the electronic supplementary material.

Supplementary file1 (DOCX 22 KB)

Supplementary file2 (DOCX 261 KB)

## Data Availability

Data will be made available from the corresponding author upon a reasonable request.

## Abbreviations

APACHE II: Acute Physiology and Chronic Health Disease Classification System II
AUC: Area under the curve
CI: Confidence interval
CAM-ICU: Confusion Assessment Method for the Intensive Care Unit
CPR: Cardiopulmonary resuscitation
DSI: Delirium screening instrument
DSM-IV-TR: Diagnostic and Statistical Manual, 4th Edition, Text Revision
GCS: Glasgow Coma Scale
ICDSC: Intensive Care Delirium Screening Checklist
ICU: Intensive care unit
NPV: Negative predictive value
PPV: Positive predictive value
RASS: Richmond Agitation Sedation Scale
ROC curve: Receiver operating characteristics curve
SAPS II: Simplified Acute Physiology Score II
SOP: Standard operating procedure
ssCAM-ICU: Severity Scale Confusion Assessment Method for the Intensive Care Unit
TIA: Transient ischemic attack
