# Supplementary material for: Influence of Patient-Specific Covariates on Test Validity of Two Delirium Screening Instruments in Neurocritical Care Patients (DEMON-ICU)
Source: Neurocrit Care. 2021 Aug 9;36(2):452–62. doi: 10.1007/s12028-021-01319-9 (PMC8351768; doi:10.1007/s12028-021-01319-9)
Supplement: Supplementary file 1 — Supplementary file1 (DOCX 22 KB) [file 12028_2021_1319_MOESM1_ESM.docx]

**Supplement 1:** Diagnoses of patients (n = 21) grouped as ‘others’ in Table 1

| **ICD-10 code** | **Diagnosis** | **Number of patients, n** |
| --- | --- | --- |
| Q07.0 | Arnold-Chiari syndrome | 1 |
| M84.8 | Disorders of continuity of bone, other | 1 |
| A32.1 | Listerial meningitis and meningoencephalitis | 1 |
| D32 | Benign neoplasm of meninges | 2 |
| D16.41 | Benign neoplasm of bone and articular cartilage, Bones of skull and face | 1 |
| T81.4 | Infection following a procedure, not elsewhere classified (sepsis) | 2 |
| S12.8 | Fracture of other parts of neck | 1 |
| T79.9 | Unspecified early complication of trauma | 1 |
| Q28.2 | Congenital arteriovenous malformation of cerebral vessels | 1 |
| G40.2 | Localization-related (focal)(partial) symptomatic epilepsy and epileptic syndromes with complex partial seizures | 1 |
| G06.2 | Extradural and subdural abscess, unspecified | 1 |
| G03.8 | Meningitis due to other specified causes | 1 |
| G40.3 | Generalized idiopathic epilepsy and epileptic syndromes | 1 |
| G61.0 | Guillain-Barré syndrome | 1 |
| G50.0 | Trigeminal neuralgia | 1 |
| G41.2 | Complex partial status epilepticus | 1 |
| G40.9 | Epilepsy, unspecified | 1 |
| G45.9 | Transient cerebral ischaemic attack, unspecified | 1 |
| G91.2 | Normal-pressure hydrocephalus | 1 |
