# Supplementary material for: Influence of Patient-Specific Covariates on Test Validity of Two Delirium Screening Instruments in Neurocritical Care Patients (DEMON-ICU)
Source: Neurocrit Care. 2021 Aug 9;36(2):452–62. doi: 10.1007/s12028-021-01319-9 (PMC8351768; doi:10.1007/s12028-021-01319-9)
Supplement: Supplementary file 2 — Supplementary file2 (DOCX 261 KB) [file 12028_2021_1319_MOESM2_ESM.docx]

**Supplement 2:** Empirical ROC curves for the **(A)** ICDSC and **(B)** ssCAM-ICU depending on the RASS score, mechanical ventilation (yes/no), neurosurgical intervention (yes/no) and acute diagnosis of stroke/TIA (yes/no) (inclusion of all assessments).

**A:** AUC depending on RASS score: 0.921 (RASS 0 or -1, n = 306); 0.619 (RASS < -1 or > 0, n = 48).

AUC depending on mechanical ventilation: 0.913 (mechanical ventilation, n = 20); 0.892 (no mechanical ventilation, n = 334). AUC depending on neurosurgical intervention: 0.898 (neurosurgical intervention, n = 204); 0.882 (no neurosurgical intervention, n = 150). AUC depending on acute diagnosis of stroke/TIA: 0.901 (acute stroke/TIA, n = 194); 0.884 (no acute stroke/TIA, n = 160). **B:** AUC depending on RASS score: 0.931 (RASS 0 or -1, n = 299); 0.619 (RASS < -1 or > 0, n = 24). AUC depending on mechanical ventilation: 0.852 (mechanical ventilation, n = 15); 0.911 (no mechanical ventilation, n = 308). AUC depending on neurosurgical intervention: 0.940 (neurosurgical intervention, n = 181); 0.853 (no neurosurgical intervention, n = 142). AUC depending on acute diagnosis of stroke/TIA: 0.899 (acute stroke/TIA, n = 180); 0.916 (no acute stroke/TIA, n = 143). AUC = Area under the curve; ICDSC = Intensive Care Delirium Screening Checklist; Mech. Vent. = Mechnical ventilation; RASS = Richmond Agitation Sedation Scale; ROC = Receiver operating characteristic; ssCAM-ICU = Severity scale Confusion Assessment Method for the Intensive Care Unit; TIA = Transient ischemic attack.
